# Effects of oral steroid sparing immunosuppressive drugs in long term maintenance treatment of chronic inflammatory demyelinating polyradiculoneuropathy

**DOI:** 10.12669/pjms.40.8.7719

**Published:** 2024-09

**Authors:** Ali Sajjad, Sajid Hameed, Sara Khan

**Affiliations:** 1 Ali Sajjad, MBBS, FCPS (Neurology) Department of Medicine, Neurology Section, Aga Khan University and Hospital Karachi, Pakistan; 2 Sajid Hameed, MBBS, FCPS (Neurology) Department of Medicine, Neurology Section, Aga Khan University and Hospital Karachi, Pakistan; 3 Sara Khan, MD. Department of Medicine, Neurology Section, Aga Khan University and Hospital Karachi, Pakistan

**Keywords:** Chronic inflammatory demyelinating polyradiculoneuropathy, CIDP, Immunosuppressive drugs (ISD), IVIG, Plasmapheresis

## Abstract

**Background and objectives:**

Chronic inflammatory demyelinating polyradiculoneuropathy (CIDP) is an acquired treatable autoimmune disorder. Due to limited availability and affordability of IV immunoglobulins and therapeutic plasma exchange in Pakistan, oral immunosuppressive drugs (ISDs) are used despite limited role in literature. The study aimed to determine the response to ISDs in CIDP patients by assessing the frequency of remission, reduction of disability using a neuropathy related disability score called Inflammatory Neuropathy Cause and Treatment score (or INCAT score), as well as reduction in steroid maintenance dose.

**Methods:**

The retrospective observational study of six months duration (May to October, 2020) was carried out in Aga Khan University Hospital, Karachi, Pakistan. Medical record of all the patients with idiopathic CIDP taking oral ISDs in last five years was selected which included bio-data, clinical signs and symptoms, medication details, and INCAT scores. Descriptive statistics were described i.e. frequency, percentages, mean/standard deviation using Microsoft Excel v.2021.

**Results:**

Out of thirteen patients, Azathioprine was used in nine, Mycophenolate mofetil in two and Cyclosporine in two, with remission (INCAT score improvement ≥ 1) achieved in eight, one and zero patients respectively. Duration of ISDs ranged from three to twenty-four months (average 15.8 months). Patients with monoclonal paraproteinemia and prior exposure to ISDs had a poor response to the introduction of subsequent ISDs.

**Conclusion:**

The study describes preliminary experience of the potential role of relatively cheaper and more convenient oral ISDs (especially Azathioprine) as an alternative or sparing agent to first line agents for CIDP and sets the stage for larger scale studies and randomized controlled trials.

**Abbreviations:**
CIDP: Chronic inflammatory demyelinating polyradiculoneuropathy,
NCAT: Inflammatory Neuropathy Cause and Treatment,
ISD: Immunosuppressive drug.
IVIG: Intravenous immunoglobulins,
MGUS: Monoclonal gammopathy of unknown significance,
POEMS: Polyneuropathy, Organomegaly, Endocrinopathy, Monoclonal gammopathy, and Skin changes.
TPE: Therapeutic plasma exchange.

## INTRODUCTION

Chronic inflammatory demyelinating polyradiculoneuropathy (CIDP) is an autoimmune disorder that affects peripheral nerves and nerve roots. The global incidence and prevalence rates of CIDP are estimated to be 0.33 and 2.81 per 100,000 person years, respectively.^1^ For Asia, the only statistics available are from a Japanese study that estimated an annual incidence of 0.48 and prevalence of 1.61 per 100,000 persons in the total population.^2^ CIDP can be a disabling disease, with at least half of the affected patients requiring assistance for daily activities.^3^

Gold standard first line treatment options include high dose steroids, intravenous immunoglobulin (IVIG) and therapeutic plasma exchange (TPE). Although limited literature exists on the effectiveness of oral immunosuppressive drugs (ISDs), e.g., methotrexate and azathioprine, as first-line treatment sparing agents, the overall results are unsatisfactory.^4^,^5^ Despite these results, ISDs are commonly used in CIDP treatment due to resistance, adverse effects, cost, or administration issues with first-line treatment options.

In Pakistan, limited data is available on CIDP with only two small retrospective studies reporting a frequency of 0.9% to 1.15% among patients undergoing electrodiagnostic examination for various indications.^6^,^7^ Due to limited availability and affordability of IVIG and TPE in our country, steroids are commonly used to achieve and sustain remission, along with oral ISDs. Our study aims to assess the effectiveness of oral ISDs as more affordable and convenient alternatives to first-line agents for maintaining remission. Therefore, the primary objective of our study was to assess ISDs response (remission/relapse) by using Inflammatory Neuropathy Cause and Treatment (INCAT) scores. The secondary objective was to reduce the baseline steroid dose by at least 50% after starting ISDs while maintaining the remission. We are optimistic that our study will also guide to conduct large prospective multi-center studies and randomized control trials (RCTs) to help us determine the potential long-term role of ISDs in managing CIDP cases.

## METHODS

This is a retrospective observational study with consecutive sampling technique. The duration of the study was six months (May 2020 to October 2020). The medical record of all the patients diagnosed with idiopathic CIDP on electrophysiological basis and taking oral ISDs presenting to the Aga Khan University Hospital, Karachi, Pakistan in previous five years (i.e. between the periods of January 2015 and June 2020) was selected. Only patients with idiopathic CIDP were included. All patients had a follow up of at least six months duration. Patient’s bio-data, clinical sign and symptoms, details of medications (dose, timing, duration and adverse effects of steroids, IVIG, TPE and oral ISDs) and INCAT scores were recorded. Data consisted mainly of descriptive information and did not require any comprehensive analysis. Therefore, data was recorded in Microsoft Excel version 2021 and no analytical software (i.e. SPSS) was utilized.

### Ethical Approval:

Ethical review committee approval (No. 2020-2096-10348) was obtained on 26^th^ April 2020. Operational definitions:

### Chronic inflammatory demyelinating polyradiculoneuropathy:

A progressive symmetrical or asymmetrical proximal or distal weakness, relapsing or progressive course >2 months, large fiber neuropathy types sensory loss (vibration and joint position sense) in the distal limbs and generalized hyporeflexia or areflexia.^8^

### INCAT Score:

The INCAT (Inflammatory Neuropathy Cause and Treatment) disability score is a measure of limitation of activity attributed to neuropathy. It was derived from Guy’s Neurological Disability Scale (GNDS) aimed for multiple sclerosis patients. The scale is highly reliable, valid and feasible for measuring disability due to neuropathy. 9

### Response/Remission:

Responders are defined as those who had a reduction (improvement) of at least one adjusted INCAT disability score point compared to their baseline.^10^

### Relapse:

Relapse is defined as an increase of at least one adjusted INCAT disability score point from baseline, except for an increase from 0 to 1 in the upper limb score.^10^

### Inclusion criteria:

All adult patients diagnosed with idiopathic CIDP by a consultant neurologist, with findings consistent with the clinical definition (as described above) and confirmed on electrophysiological testing based on American Academy of Neurology Ad Hoc Subcommittee Electrodiagnostic criteria^11^ and taking oral ISD for remission were included. However, due to strong association of monoclonal gammopathy of unknown significance (MGUS) with CIDP without any direct neuropathic effects of these monoclonal antibodies, two such cases were included in the study. Patients with MGUS underwent extensive hematological work up and were found negative for multiple myeloma and POEMS (Polyneuropathy, Organomegaly, Endocrinopathy, Monoclonal gammopathy, and Skin changes) syndrome.

### Exclusion criteria:

All patients with associated co-morbids that can potentially cause a neuropathy e.g. diabetes mellitus, vitamin B12 deficiency, co-existing malignancy / paraneoplastic disease, systemic autoimmune diseases, multifocal motor neuropathy, mono-neuritis multiplex or hereditary sensorimotor polyneuropathy, were also excluded based on their history, examination and electrophysiological findings. Additionally, patients less than 18 years of age or with prolonged neurological deficits due to stroke or other central nervous system disorders were excluded.

## RESULTS

Thirteen CIDP patients were included, with a mean age of 48.1 ± 13.1 years (range: 30-72 years), with a male predominance (84.6%, n=11). Relapsing remitting (53.8%, n=7) was the most common CIDP type seen. Duration of CIDP, from symptom onset to diagnosis, ranged from 7-28 months (mean 15.8 ± 6.8 months) (Table-I). All patients, except one, received steroids (oral prednisolone) at some point during their illness. Two cases (9 and 10) had an IgG lambda-type monoclonal gammopathy of unknown significance (MGUS).

**Table-I:** Clinical features and treatment regimens of patients with CIDP.

Cases	Current ISDs	Age range (years)	Disease duration (months)	Symptoms	Clinical features	Prednisolone (Ever used)	Prior history of IVIG or TPE	Timing of IVIG / TPE before/with ISDs	Prior ISD duration in months	Range of daily dose (mg/day)	Duration of ISDs (months)
1	Azathioprine	60	16	S	RRMS	Yes	No	No	No	50-100	16
2	Azathioprine	30	28	SM	RRMS	Yes	1 session IVIG	8 months prior	No	100-150	20
3	Azathioprine	49	27	SM	RRMS	Yes	1 session TPE 1 session IVIG	4 months prior 3 months prior	No	100-150	4
4	Azathioprine	72	09	M	RRMS	Yes	No	No	No	50-150	6
5	Azathioprine	38	24	SM	Mono	Yes	1 session IVIG	Given at the start of course	No	50-100	24
6	Azathioprine	53	13	SM	Mono	Yes	No	No	No	100-150	5
7	Azathioprine	39	12	SM	Mono	Yes	No	No	No	50-100	12
8	Azathioprine	33	12	SM	RRMS	Yes	No	No	No	50-100	12
9	Azathioprine*	38	12	SM	RRMS	Yes	No	No	Cyclosporine for 2-3 month (no improvement)	100-150	9
10	Cyclosporine*	36	16	M	Mono	Yes	1 session IVIG	Given at the start of cyclosporine	Azathioprine 50-100 mg/day for 8 months (no improvement) needed IVIG	50	3
11	Cyclosporine	67	7	M	Mono	Yes	No	No	No	150	7
12	Mycophenolate Mofetil	49	22	M	RRMS	Yes	3 sessions IVIG	2 month prior	Azathioprine 50-150 mg/day for 6 months (no improvement; needed IVIG)	1000 – 1500	14
13	Mycophenolate Mofetil	61	8	M	Mono	No	1 session TPE	Given at the start of course	No	1000-2000	8

CIDP – chronic inflammatory demyelinating polyradiculoneuropathy, ISDs – Immunosuppressive drugs, TPE – therapeutic plasma exchange, IVIG – IV immunoglobulins, RRMS – Relapsing remitting MS, Mono – Monophasic, S- sensory, SM – sensorimotor, M - Motor

* Cases with IgG lambda monoclonal gammopathy).

Duration of ISDs ranged from 3-24 months (mean 15.8 ± 6.18 months). Among thirteen cases, the majority (69.2%, n=9) received azathioprine, followed by cyclosporine and mycophenolate mofetil in two cases each (15.4%). Three patients changed their ISD during the course of illness due to insufficient improvement with the initial ISD (azathioprine in two cases, cyclosporine in one). The daily dose range and duration of different ISDs are provided in Table-I. Prior to or at the start of ISDs, six patients received either IVIG or TPE, while one patient received both IVIG and TPE.

All patients receiving azathioprine except one (89%, n=8 out of nine) were able to achieve remission and were also able to reduce baseline steroid dose by at least 50%, with steroids completely stopped in five patients (55% patients n=5 out of 9), without CIDP relapse indicating a generally favorable response (Table-II). On the contrary, both patients receiving cyclosporine (case # 10 and 11) were unable to achieve remission or taper off oral prednisolone and continued to have worsening symptoms and one patient required IVIG. Partial response (i.e. 50%, n= 1 out of two) was noted with mycophenolate mofetil. One patient using the medication remained in remission without the need for steroids after an initial single course of TPE, while the other patient did not achieve remission despite previously receiving multiple treatments of IVIG and azathioprine.

**Table-II:** Clinical course of the disease on ISDs and side-effects.

Cases	Current ISDs	Prednisolone dose tapering (mg/day)	Able to reduce DC by ≥ 50%	INCAT Changes	Improvement/ Worsening	Given IVIG / TPE despite IM	Side-effects
1	Azathioprine	20 to stop	Yes	0 to 0	S improvement	No	Nil
2	Azathioprine	60 to stop	Yes	3 to 1	S+M improvement	No	Nil
3	Azathioprine	60 to 30	Yes	4 to 2	S+M improvement	No	Disseminated Nocardiosis
4	Azathioprine	30 to 7.5	Yes	2 to 0	M improvement	No	Steroid induced psychosis
5	Azathioprine	60 to stop	Yes	2 to 0	S+M improvement	No	Nil
6	Azathioprine	60 to stop	Yes	4 to 0	S+M improvement	No	Nil
7	Azathioprine	30 to stop	Yes	3 to 0	S+M improvement	No	Mild hepatotoxicity (underlying fatty liver)
8	Azathioprine	60 to stop	Yes	3 to 0	S+M improvement	No	Nil
9	Azathioprine*	30 to 10	No (relapse on taper)	1 to 3	Both SM worse	No	Nil
10	Cyclosporine*	40 to 40	No	4 to 5	M worse	No	Nil
11	Cyclosporine	60 to 60	No (relapse on taper)	5 to 6	M worse	IVIG (1 session) After 7 months of cyclosporine	No
12	Mycophenolate Mofetil	30 to 30	No (relapse on taper)	1 to 2	M worse	No	Nil
13	Mycophenolate Mofetil	Not used at all	NA	2 to 0	M improvement	No	Nil

CIDP = chronic inflammatory demyelinating polyradiculoneuropathy; INCAT = Inflammatory Neuropathy Cause and Treatment; ISDs = Immunosuppressive drugs; IVIG = Intravenous immunoglobulins; M= Motor; NA= Not applicable; S= Sensory; TPE – therapeutic plasma exchange

* (Cases with IgG lambda monoclonal gammopathy)

Regarding adverse effects, ISDs were generally well-tolerated. Out of nine patients receiving azathioprine, one patient developed systemic Nocardiosis. This patient was also on oral steroids. Transaminitis was observed in one patient with a history of underlying fatty liver disease. The liver enzyme levels normalized after reducing the azathioprine dose from 100 mg/day to 50 mg/day. One patient developed psychosis secondary to steroid use (not a direct azathioprine side effect) requiring a rapid tapering of steroids without clinical relapse. We did not observe any side effects with patients using cyclosporine or mycophenolate mofetil.

## DISCUSSION

CIDP can be a disabling condition but is a treatable one. The treatment, however, imposes a substantial physical and financial burden on patients. A single course of five-day TPE (40-50ml/kg/day) costs approximately $4000 to $5000. IVIG treatment (2gm/kg/course) costs around $7500 to $8000. These invasive treatments need to be repeated every few weeks to months depending on symptom severity. A Canadian study found that the annual maintenance cost for IVIG exceeded $70,000^8^, with the total cost over two years ranging from $111,406 to $117,233, which was significantly higher compared to steroids alone ($3101).^12^ A U.S. based study reported that more than half of their newly diagnosed CIDP patients were taking oral steroids only.^12^ Given the average monthly income of $170 and lack of insurance coverage in our country, most of our CIDP patients are on a “steroid-only” treatment plan, with or without oral ISDs. In addition to the cost of oral ISDs, there is an additional expense for regular hematological and liver function tests but still ISDs remain relatively affordable, with azathioprine costing around $150 annually.^13^

Existing literature is limited regarding the therapeutic effectiveness of ISDs in CIDP and presents conflicting evidence. Our experience with azathioprine (1-2.5 mg/kg/day) has been encouraging. We observed positive responses in majority of our patients (89%) and in some patients that response continued for up to 24 months, which is considerably longer than other studies.^4^ The one patient who did not respond well to azathioprine also had MGUS paraproteinemia (case no. 9). A RCT by Dyck et al. in 1985 compared prednisolone alone to prednisolone plus azathioprine treatment, showing no significant change in disability scores.^4^ A case series involving two patients treated with azathioprine found no benefits^14^, while other studies reported substantial improvement in a range of 35% to 95% of patients.^15^,^16^ Azathioprine treatment durations in the literature vary from 19 days to 12 months.^14^,^17^

Conversely to azathioprine, we observed unfavorable and mixed responses with cyclosporine and mycophenolate mofetil, respectively. In two CIDP patients receiving cyclosporine, steroid tapering was unsuccessful for both. One of them had MGUS paraproteinemia and also failed previous trials of azathioprine as well as one IVIG treatment session, suggesting a resistant case (case no. 10). It is worthwhile to note that our study had a short follow-up duration (3-7 months) and lower cyclosporine dosage (50-150 mg/day) compared to other studies.^14^,^17^ In case of mycophenolate mofetil, remission was maintained in one patient after IVIG without steroids while symptoms continued to worsen in the second patient despite steroids. The typical treatment duration reported in literature is 12-15 months, with a mycophenolate mofetil dose range of 1-2 gm/day, similar to our cases.^14^,^17^

Existing literature regarding cyclosporine and mycophenolate mofetil is scarce and limited to a few case series showing response rates varying from 25-90%.^14^,^17^,^18^-^20^ The largest study reported to-date observing cyclosporine effects on CIDP patients included only 19 CIDP patients.^20^ Therefore, it will be premature to conclude cyclosporine or mycophenolate mofetil a failure based on our limited data. Larger trials with higher cyclosporine doses are needed to validate its effects.

It is interesting to note that both of our CIDP patients with MGUS paraproteinemia (cases no. 9 and 10) continued to worsen despite receiving azathioprine and cyclosporine. Although this is insufficient evidence to make a conclusion, it is possible that CIDP secondary to paraproteinemia may be more resistant to treatment with ISDs. Further investigation is needed to determine the optimal treatment options for this specific CIDP subcohort. Generally, 66-80% of patients with CIDP associated with monoclonal gammopathy respond to corticosteroids, IVIG, TPE, and ISD (including azathioprine and fludarabine)^21^, with a greater disease progression compared to idiopathic CIDP on long-term follow-up.^22^

### Limitations:

This is a limited single-center observational study with a small sample size and short duration. Admittedly, the variability of the clinical course of CIDP itself may confound the response to ISDs. Larger observational and interventional studies with longer durations are required at the national and international level to create more reliable inferences.

## CONCLUSIONS

CIDP is a rare, potentially disabling but treatable disorder. Our study aimed to provide preliminary insights into the use of cost-effective and convenient oral ISDs, particularly azathioprine, as long-term steroid-sparing drugs in CIDP management. Although we have tried to fill some gaps in the existing literature and share our local experience, we still need larger local studies and RCTs to confirm the potential of oral ISDs as first-line steroid-sparing agents. Furthermore, CIDP patients with MGUS and prior exposure to a different ISD showed a limited response to treatment.

### Author`s Contribution:

**AS:** Acquisition, analysis and interpretation of data and drafting of the article. Responsible and accountable for the accuracy and integrity of the work.

**SH:** Drafting and critical revision of the article.

**SK:** Conception and design of the study, critical revision of the article.
